# Comparison of patient-reported outcomes between alternative care provider-led and physician-led care for severe sleep disordered breathing: secondary analysis of a randomized clinical trial

**DOI:** 10.1186/s41687-024-00747-3

**Published:** 2024-09-26

**Authors:** Maria J. Santana, Oyindamola Jaja, Qiuli Duan, Erika D. Penz, Kristin L. Fraser, Patrick J. Hanly, Sachin R. Pendharkar

**Affiliations:** 1 Department of Pediatrics, Cumming School of Medicine, University of Calgary, Calgary, Alberta Canada; 2 Department of Community Health Sciences, Cumming School of Medicine, University of Calgary, Calgary, Alberta Canada; 3 Alberta Strategy for Patient-Oriented Research, Calgary, Alberta Canada; 4 Shoppers Drug Mart, Lethbridge, Alberta Canada; 5 Data & Analytics, Alberta Health Services, Calgary, Alberta Canada; 6 Division of Respirology, Critical Care and Sleep Medicine, University of Saskatchewan, Saskatoon, Saskatchewan Canada; 7 Respiratory Research Centre, University of Saskatchewan, Saskatoon, Saskatchewan Canada; 8 Department of Medicine, Cumming School of Medicine, University of Calgary, Calgary, Alberta Canada; 9 Foothills Medical Centre Sleep Centre, University of Calgary, Calgary, Alberta Canada; 10 Hotchkiss Brain Institute, Cumming School of Medicine, University of Calgary, Calgary, Alberta Canada

**Keywords:** Sleep medicine, Patient reported outcomes, Health quality improvement, Models of care, Patient access

## Abstract

**Background:**

Previous research has suggested that alternative (respiratory) care providers (ACP) may provide affordable, accessible care for sleep-disordered breathing (SDB) that decreases wait-times and improves clinical outcomes. The objective of this study was to compare ACP-led and sleep physician-led care for SDB on patient reported outcome and experiences, with a focus on general and health-related quality of life, sleepiness, and patient satisfaction.

**Methods:**

We conducted a secondary analysis of a randomized trial in which participants with severe SDB were assigned to either ACP-led or physician-led management. We created longitudinal linear mixed models to assess the impacts of treatment arm and timepoint on total and domain-level scores of multiple patient-reported outcome measures and patient-reported experience measures.

**Results:**

Patients in both treatment arms (ACP-led n = 81; sleep-physician = 75) reported improved outcomes on the Sleep Apnea Quality of Life Index, Health Utilities Index, and Epworth Sleepiness Scale. Patients in each group had similar and clinically meaningful improvements on domains assessing cognition, emotion, and social functioning. The linear mixed models suggested no significant difference between treatment arms on the patient-reported outcomes. However, scores significantly improved over time.

**Conclusions:**

Management of SDB using ACPs was comparable to physician-led care, as measured bypatient-reported outcome and experience measures. While loss to follow-up limits our findings, these results provide some support for the use of this novel health service delivery model to improve access to high quality SDB care.

**Clinical trial registration:**

This is analysis of data from the study registered Clinicaltrials.gov (NCT02191085).

**Supplementary Information:**

The online version contains supplementary material available at 10.1186/s41687-024-00747-3.

## Introduction

Sleep-disordered breathing (SDB) comprises several entities including obstructive sleep apnea (OSA), central sleep apnea and sleep related hypoventilation, of which OSA is the most common. In Canada, it is estimated that 30% of adults have a moderate to high likelihood of having OSA, but only 6.4% have been diagnosed [1]. Untreated SDB is associated with excessive daytime sleepiness, depression, increased risk of motor vehicle accidents, significant cardiovascular morbidity, and death [2]. Economic impacts of SDB range from a loss of productivity in the workplace to increased health services utilization [3–5]. The treatment of SDB includes the use of positive airway pressure (PAP) either alone or in combination with supplemental oxygen. This treatment is cost effective and has been reported to reduce daytime symptoms, automobile crashes, cardiovascular risk, and healthcare utilization [2].

Delayed access to sleep care has been reported in Canada, Australia, Europe and the USA [6, 7]. One strategy to improve timely access involves the use of trained non-physician alternative care providers (ACPs) such as nurses or respiratory therapists in the management of SDB [8, 9]. ACP-led models allow for reallocation of staff and resources that may ultimately improve time-to-treatment [10], costs to the healthcare system [11], and patient experiences of care [12].

Patient-reported outcome measures (PROMs) and experience measures (PREMs) play a key role in identifying outcomes that are important to patients and to understanding how patients experience care [13–15]. Patient-reported measures such as the Health Utilities Indexes, the 12 and 36 Item Short Form Health Survey (SF-12 and SF-36), Epworth Sleepiness Scale, and the Sleep Apnea Quality of Life Index have been used to evaluate patient outcomes in sleep medicine [16]. PROMs have been used as part of evaluations of clinical interventions in sleep medicine (such as in the comparison of CPAP with oral appliances [17]), and PROMs are sensitive to changes in treatment [18]. Furthermore, domain-specific analyses can provide richer insights than more conventional measures on outcomes that are relevant and important to patients [19, 20]. Beyond the biological indicators of disease status and improvement, incorporating patient-reported measures to evaluate alternative models of care can allow policymakers and clinicians to understand their impact on patients.

The objective of the present study was to examine longitudinal differences over 12 months in PROMs and PREMs between ACP-led and sleep physician-led care for SDB. Based on prior results comparing baseline and 3-month follow-up [12] we expected that patients who received ACP-led care would have greater improvements in sleepiness, quality of life, and satisfaction compared to those who received sleep physician-led care.

## Methods

### Study design

This is a secondary analysis of a randomized, controlled, non-inferiority trial comparing the management of severe SDB (severe OSA or hypoventilation) by ACPs supervised by sleep physicians to standard management by sleep physicians alone [12]. Ethics approval was obtained from the Conjoint Health Research Ethics Board (Ethics ID: REB13-1280) at the University of Calgary.

### Study setting and eligibility criteria

The protocol of the full study has been previously published. Patients were recruited following referral to the Foothills Medical Centre (FMC) Sleep Centre for assessment of SDB. The FMC Sleep Centre is a publicly funded, tertiary academic sleep centre in Calgary, Alberta and receives approximately 2500 referrals annually; 90% of referrals are for SDB and 30% of referrals are for severe SDB [21]. At the FMC Sleep Centre, ACPs are respiratory therapists who have graduated from accredited three-year programs, are registered with the provincial governing body (College & Association of Respiratory Therapists of Alberta, CARTA), and have at least five years of experience in managing SDB. Patients were included in the study if they met at least one of the following inclusion criteria: respiratory event index (REI) ≥ 30 events/hour on home sleep apnea testing (HSAT), mean nocturnal oxygen saturation ≤ 85% on HSAT, suspected sleep hypoventilation syndrome defined by a partial pressure of carbon dioxide ≥ 45 mmHg on arterial blood gas analysis accompanied by an REI ≥ 15 events/hour on HSAT. Patients were excluded if they had a suspected concomitant sleep disorder other than SDB such as insomnia, had previously been treated with PAP therapy for SDB, had primary health insurance from outside Alberta, or did not provide consent to participate in the study.

### Study procedure

Briefly, patients were randomized to receiving either ACP-led care or sleep physician-led care. In the ACP arm, practitioners worked with patients to develop care plans, with review by a supervising sleep physician. In the sleep physician arm, physicians developed care plans with patients. In both arms, sleep physicians were responsible for ordering and interpreting sleep diagnostic tests and providing treatment prescriptions. A detailed description of the procedure can be found in the publication of the trial protocol [8].

### Data sources

Relevant clinical and patient-reported data were collected at baseline, 3 months, and 1 year after treatment initiation. Baseline study data were collected from referral information (patient demographics), HSAT (BMI, respiratory event index), polysomnogram (apnea hypopnea index) and by self-report (comorbidities, medications). Treatment adherence was collected from machine downloads stored on a digital card or in cloud-based storage maintained by the device manufacturer.

### Study outcomes

In this secondary analysis, we compared overall patient reported outcomes and experience (visit satisfaction), as well as measurement domains for each instrument between ACP-led care and physician-led care. Patient-reported outcomes and experiences were determined using general and disease specific instruments at baseline, 3 months, and 12 months after treatment initiation (with the exception of the Visit Specific Questionnaire (VSQ-9), which was completed only at 3 months and 12 months). The Health Utility index (HUI [19]) Mark 2 and 3 were used to measure general health-related quality of life (HRQoL), the Sleep Apnea Quality of Life Index (SAQLI [22]; was used to measure disease-specific HRQoL, and Epworth Sleepiness Scale (ESS [23]); was used to measure symptoms at baseline, 3 months and 1 year.

The HUI measures have been used in a variety of clinical settings to provide comparable patient-reported assessment of health status and quality of life across several attributes (HUI-2: sensation, mobility, emotion, cognition, self-care, pain, fertility; HUI-3: vision, hearing, speech, ambulation, dexterity, emotion, cognition, pain) [19]. Utility scores on the HUI-2 range from −0.03 to 1.00; scores on the HUI-3 range from −0.36 to 1.00 [19]. A 1.00 denotes perfect health and a 0 denotes death; scores lower than 0 allow for “health states considered worse than dead” [19]. Meaningful improvements on the HUI scores are reflected by score improvements of 0.05 for the overall HUI score and 0.05 for improvement within attribute (domain) scores [19]. Normative values for Canadians (aged 12+) are 0.863 for the HUI-3 [19, 24]. In one study reporting on a sample of patients at a sleep centre, mean scores were 0.73 for the HUI-2 and 0.60 for the HUI-3 [25].

The short form of the Sleep Apnea Quality of Life Index (SAQLI) is a 14 item patient-reported outcome measure that assesses HRQoL specific to OSA and its treatment [22, 26]. Respondents rate a series of questions on daily functioning, social interactions, emotional functioning, and symptoms on a seven-point scale with labels ranging from “all the time” to “not at all”. Domain-specific scores are calculated by averaging the domain-specific questions and total score is calculated the average of the domain scores [26]. We also used the Epworth Sleepiness Scale, an eight-question patient-reported outcome measure that evaluates the likelihood of falling asleep in various activities of daily life [23, 27, 28]. Additional information on the HUI, SAQLI, and ESS can be found in the supplementary file.

Patient-reported experiences were measured using the Visit-Specific Satisfaction (VSQ-9) which is a validated measure of patient experience with an outpatient visit [29]. The VSQ-9 asks participants to rate nine elements of the visit experience (e.g. wait times for appointment, provider skill and communication, overall experience) from 1 (“poor”) to 5 (“excellent”).

### Statistical analysis

Descriptive analysis was used to summarize baseline patient characteristics. Outcomes data from the primary study were recoded to generate each patient’s ESS total scores, individual domain scores for HUI-2 (Sensation, Mobility, Emotions, Cognitive, Selfcare, Pain), HUI-3 (Vision, Hearing, Speech, Ambulation, Dexterity, Emotion, Cognition, Pain), and SAQLI (Symptom, Emotion, Social interaction, Daily activity) at baseline, 3 months and 1 year. VSQ-9 total scores were recorded at 3 months and 1 year. Since observations were measured at multiple times, Linear Mixed Models (LMM) were constructed to examine if there was a difference in these patient-reported outcomes over the three time points and between the ACP-led clinic and physician-led care. No imputation was performed for missing data. Patients needed to have completed the ESS to be included for analysis. Statistical analysis was done using SAS Enterprise Guide 7.1 (Cary, NC).

## Results

**Fig. 1:**
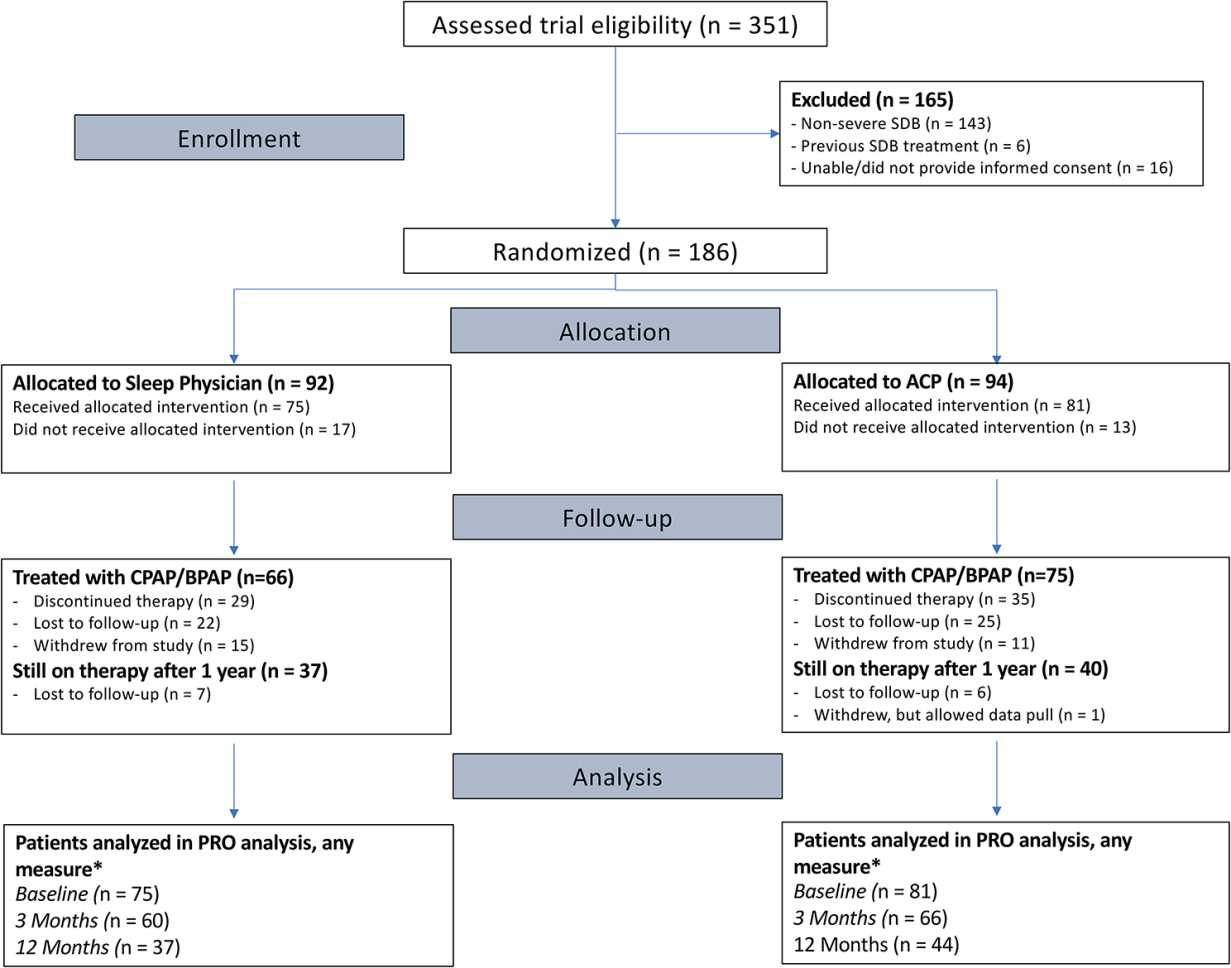
CONSORT diagram for the study. *Patients needed to have completed the ESS measure to be eligible for analysis in this study

Patient characteristics at baseline are included in Table 1. Some of these descriptive statistics for the 156 patients have been previously published. As reported in Pendharkar et al., the average age for the total cohort was 54.5 years (standard deviation: 12 years) and the average BMI was 39 kg/m^2^ (standard deviation: 9.8 kg/m^2^). Dropout rates were comparable between groups. For the ESS, SAQLI, and HUI, 37 (45.7%) patients were lost in the ACP-led arm and 38 (50.7%) patients were lost in the sleep-physician-led arm. For the VSQ-9, 48 (53.3%) were lost in the ACP-led arm and 40 (53.3%) were lost in the sleep physician arm. Figure 1 shows the CONSORT diagram for this study.

**Table 1:** Baseline characteristics

Variable	Treatment arm	
	ACP-led care (N = 81)	Sleep physician-led care (N = 75)
**Age (mean, SD)**	54 (12)	55 (13)
**Sex (n, %)**		
Female	23 (28.4)	21 (28.0)
**BMI, kg/m**^**2**^** (mean, SD)**	40 (10)	39 (9)
**Comorbidities (n, %)**		
Hypertension	47 (63)	49 (60)
Diabetes	21 (28)	21 (26)
Cardiovascular disease	16 (21)	15 (19)
Chronic lung disease	16 (21)	23 (28)
Chronic kidney disease	5 (7)	0
**Smoking history (n, %)**		
Never	31 (38.27)	28 (37.33)
Current	23 (28.40)	12 (16.00)
Former	27 (33.33)	35 (46.67)
**REI ≥ 30 (n, %)**	67 (82.72)	63 (84.00)
**HSAT, Sp**_**O2**_** (mean, SD)**	86 (5)	85 (5)

Results are presented as mean (%) unless otherwise stated

*ACP* Alternative Care provider-led care, *HSAT*  home sleep apnea testing, *REI* Respiratory Event Index on home sleep apnea testing

### Health utilities index

Results for the HUI overall and domain scores at baseline, 3 months and 12 months are presented in Table 2 and linear mixed models evaluating effect of time and treatment arm are reported in Table 3. Overall utility scores on the HUI improved over time for both the Mark 2 (HUI-2; *F*: 10.72, *p* < 0.0001) and Mark 3 (HUI-3; *F*: 20.27, *p* < 0.0001) indexes.

**Table 2:** Changes between baseline and follow-up on patient-reported outcome measures

Measurement		Baseline	3-Month	12-Month	Difference in mean (12-month– baseline)
PROM	Domain/Attribute				
HUI2	Overall	0.73 (0.15)	0.8 (0.19)	0.81 (0.21)	0.09*
	Emotion	0.89 (0.14)	0.92 (0.13)	0.95 (0.09)	0.06*
	Cognition	0.91 (0.09)	0.95 (0.08)	0.95 (0.09)	0.04*
	Pain	0.83 (0.29)	0.89 (0.20)	0.88 (0.21)	0.05*
	Mobility	0.93 (0.12)	0.93 (0.13)	0.93 (0.14)	0
	Sensation	0.80 (0.23)	0.83 (0.20)	0.85 (0.20)	0.05
	Self-care	0.99 (0.04)	0.98 (0.10)	1 (0.02)	0.01
HUI3	Overall	0.58 (0.31)	0.70 (0.30)	0.71 (0.30)	0.13*
	Hearing	0.89 (0.26)	0.93 (0.22)	0.93 (0.22)	0.04
	Speech	0.97 (0.08)	0.98 (0.09)	0.98 (0.09)	0.01
	Ambulation	0.89 (0.16)	0.90 (0.17)	0.90 (0.18)	0.01
	Dexterity	0.98 (0.08)	0.97 (0.11)	0.97 (0.09)	−0.01
	Emotion	0.87 (0.18)	0.91 (0.15)	0.94 (0.11)	0.07*
	Cognition	0.86 (0.19)	0.93 (0.14)	0.92 (0.16)	0.06*
	Pain	0.78 (0.27)	0.85 (0.20)	0.84 (0.20)	0.06*
SAQLI	Overall	4.52 (1.37)	5.51 (1.24)	5.71 (1.20)	1.19*
	Symptoms	12.68 (5.17)	7.7 (4.22)	7.09 (4.17)	−5.59
	Emotional functioning	8.91 (4.53)	6.65 (3.51)	6.23 (3.52)	−2.68
	Social interactions	12.56 (5.71)	8.12 (4.31)	7.02 (3.6)	−5.54
	Daily functioning	14.59 (6.38)	9.83 (4.9)	9.21 (4.99)	−5.38
ESS	ESS Total	10.76 (5.62)	6.08 (4.64)	5.29 (4.15)	−5.47
VSQ-9	VSQ-9 Sum	–	36.13 (7.16)	36.30 (5.90)	0.18

*Indicates a statistically significant main effect of timepoint in the linear-mixed model analyses

**Table 3:** Effect of treatment arm and timepoint for the Health Utilities Indexes

Health Utilities Indexes - Type 3 Tests of Fixed Effects				
Version	Domain	Effect	F value	Pr > F
Mark 2	Overall	Treatment arm	1.86	0.17
		Timepoint	10.72	<0.0001
	Emotion	Treatment arm	0.11	0.74
		Timepoint	8.46	<0.001
	Cognition	Treatment arm	2.05	0.15
		Timepoint	9.79	<0.0001
	Pain	Treatment arm	1.09	0.30
		Timepoint	3.58	0.03
	Mobility	Treatment arm	0.04	0.85
		Timepoint	0.47	0.63
	Sensation	Treatment arm	1.28	0.26
		Timepoint	2.56	0.08
	Self-care	Treatment arm	0.16	0.69
		Timepoint	1.31	0.27
Mark 3	Overall	Treatment arm	1.67	0.20
		Timepoint	20.27	<0.0001
	Emotion	Treatment arm	1.79	0.18
		Timepoint	7.46	<0.001
	Cognition	Treatment arm	1.81	0.18
		Timepoint	10.8	<0.0001
	Pain	Treatment arm	2.64	0.11
		Timepoint	6.38	<0.01
	Vision	Treatment arm	0.38	0.54
		Timepoint	0.82	0.44
	Hearing	Treatment arm	2.01	0.16
		Timepoint	1.61	0.20
	Speech	Treatment arm	2.77	0.10
		Timepoint	0.01	0.99
	Ambulation	Treatment arm	0.49	0.49
		Timepoint	0.37	0.69
	Dexterity	Treatment arm	0.39	0.53
		Timepoint	0.73	0.48

Time-dependent improvements were observed in the entire cohort for the HUI-2 domains related to emotion (assessing distress and anxiety [19]) (*F*: 8.46, *p* < 0.001) cognition (assessing learning; *F*: 9.79, *p* < 0.0001) and pain (frequency, control; *F*: 3.58, *p* < 0.03). There was no significant time-dependent effect on the sensation, mobility, or self-care domains within the HUI-2. No differences in HUI-2 domain scores were observed between treatment arms.

Within the HUI-3, the emotion domain (assessing “happiness versus depression” [19]) improved over time (*F*: 7.46, *p* < 0.001), as did the cognitive domain (assessing the “solving of day-to-day problems”; *F*: 10.8, *p* < 0.0001) and the pain domain (assessing severity; *F*: 6.38, *p* < 0.003). Improvements were observed in the entire study cohort with no between-group differences. Neither timepoint nor study arm had a significant effect on domains assessing vision, hearing, speech, ambulation, and dexterity.

There were clinically meaningful improvements on the HUI-2 (0.09) and HUI-3 (0.13) overall scores, as well as within attribute domains for emotion, pain, and cognition [19].

### Sleep Apnea Quality of Life Index

The overall score on the SAQLI improved over time (Tables 2 and 4). Over time, each of the four domains within the SAQLI showed improvement: daily functioning (*F*: 57.6; *p* < 0.0001); social interactions (*F*: 63.25 *P* < 0.0001), emotional functioning (*F*: 34.53, *P* < 0.0001), and symptoms (*F*: 93.24, *p* < 0.0001). There were no significant differences between treatment arms. The overall average SAQLI score change was 1.19 points, reflecting a small but clinically significant improvement in quality of life [20, 26].

**Table 4:** Effect of treatment arm and timepoint for the Sleep Apnea Quality of Life Index (SAQLI)

Sleep Apnea Quality of Life Index - Type 3 Tests of Fixed Effects			
Domain	Effect	F value	Pr > F
Overall	Treatment arm	0.03	0.87
	Timepoint	81.51	<0.0001
Daily activities	Treatment arm	0.1	0.94
	Timepoint	57.6	<0.0001
Social interactions	Treatment arm	0.01	0.93
	Timepoint	63.25	<0.0001
Emotional functioning	Treatment arm	0.01	0.9057
	Timepoint	34.53	<0.0001
Symptoms	Treatment arm	0.17	0.68
	Timepoint	93.24	<0.0001

### Epworth Sleepiness Scale

Scores in the Epworth Sleepiness Scale decreased over 12 months (*F*: 74.35, *p* < 0.001) with no effect of treatment arm (*F*: 1.86; *p* = 0.17). The average total decrease in ESS score was 5.47 points (Table 2), reflecting a clinically meaningful decrease in patient-reported sleepiness [30].

### Visit satisfaction

Visit experience as measured by the VSQ-9 did not differ by treatment arm (F: 3.56, *p* ≤ 0.06) or timepoint (*F*: 0.81; *p* ≤ 0.37).

## Discussion

In this secondary analysis of data from a randomized clinical trial evaluating ACP management of SDB, we found that general QoL, disease-specific QoL and patient-reported sleepiness improved over a year of treatment and did not differ between sleep physician-led versus ACP-led care. Furthermore, domain analysis for the HUI and SAQLI revealed clinically meaningful improvements in cognition, emotion, and social functioning over time that was similar between treatment arms. These results support the use of ACP-led treatment models for SDB. To our knowledge, this is the first study to explore domain-specific PROMS to evaluate models of SDB care.

Alternative models of care for SDB have the potential to improve outcomes, reduce time-to-treatment, and limit strain on the healthcare system [9–12]. In our initial randomized clinical trial, we reported that participants receiving ACP-led care had greater improvements in some patient-reported outcome measures at three months [12]. However, in this secondary analysis we found time-dependent improvements in these measures through one year after treatment initiation, both overall and for several attribute domains. We did not demonstrate differences between treatment arms, supporting non-inferiority of ACP-led care. This discrepancy may be due to the use of more sophisticated models, indicate that initial benefits of ACP-led care are superseded by primary effects of PAP therapy over time, or reflect a lack of specificity for SDB experiences in the PREM. Importantly, comparable improvements in patient reported outcomes and experiences suggest that patients found the ACP model acceptable and provide support for its adoption.

Our results reflect the positive impact that receiving treatment for sleep-disordered breathing has on specific elements of patients’ quality of life. Generally, poor sleep quality is associated with cognitive [31–33], social [34], and emotional [32, 34, 35] impairement. Research in SDB populations have also found that patients demonstrate cognitive and emotional symptoms [36, 37]. We found that treatment led to clinically meaningful improvements on both the HUI-2 and HUI-3 quality of life domains for cognition and emotion in both treatment groups [19]. Scores on the SAQLI domains for emotional and social functioning also improved. Improvements in sleepiness after treatment were consistent with previous research [9].

Patients in both treatment arms also reported significant improvements in physical quality of life. We observed clinically and statistically significant improvements in the HUI (−2 and −3) pain attributes across treatment arm. Further, there were significant improvements in the symptom and daily functioning domains within the SAQLI. We did not observe significant changes on HUI attributes for vision, hearing, speech, ambulation, dexterity, sensation, or self-care. This result may suggest that either SDB does not impact patients on these domain issues or that treatment (either ACP-led or physician-led) did not have an effect on these contributors to QOL.

There are a range of generic PROMs that have been used in SDB research beyond the HUI, including the SF-12, SF-36, and Hospital Anxiety and Depression Scale [16, 17, 25]. The HUI-2 and HUI-3 were not initially developed for applications in SDB but have been adapted for multiple chronic health conditions and validated for use in sleep medicine [19, 25]. Clinicians and administrators seeking to adopt PROMs and PREMs would benefit from additional comparison between the variety of PROMS used to measure general QoL with disease-specific PROMs (such as the SAQLI, the Quebec Sleep Questionnaire, or the Patient-Reported Apnea Questionnaire [38, 39]; Sheats discussed 5 adult disease-specific patient-reported HRQoL instruments in an article introducing clinicians to QoL measures. The author noted that health related quality of life is distinct from patient-reported sleepiness and that both have utility for clinicians treating patients with OSA [16]. Though the HUI was not featured in their review, validation studies have shown its applicability in both general and sleep-specific settings [19, 25]. Since the release of the Institute for Healthcare Improvement’s Triple Aim Framework, health systems have increasingly measured the quality of healthcare delivery by assessing the patient experience [40]. To our knowledge, there are no widely used PREMs that are specific to SDB. When designing this trial, we chose to use the VSQ-9 because it was a simple and validated visit-specific patient-reported experience measure with less measurement burden than other instruments and has been used previously in the measurement of patient experience with SDB care [11, 41, 42].

There are several strengths to this study. First, it provides evidence around the use of several PROMs and is a basis from which to approach further study of patient reported outcomes and experiences within SDB care. The paper provides unique analyses of patient-reported outcomes and experiences; to our knowledge, this is a novel application of PROMs to evaluate SDB care. The addition of longitudinal patient-reported outcome and experience data allow us to draw stronger conclusions about the acceptability of ACP clinics to patients as an alternative to the traditional physician-led model of care.

This study also has important limitations. First, study patients were heterogenous, with variable diagnoses (severe OSA vs OSA with hypoventilation) who followed different pathways (e.g., diagnosis using HSAT vs. polysomnography) and received different types of PAP therapy (continuous or bilevel PAP with or without oxygen); therefore, they may have had different experiences with care. Given the sample size, it was not feasible to compare patients within these subgroups, but these testing pathways and treatments were well-balanced between groups. Second, patient experience was only measured using satisfaction related to visits. Future work could explore patient-provider communication or person-centredness of care (e.g., involvement in care decisions). Third, this was a single-centre study of individuals with severe SDB, and the results may not be generalizable beyond our institution or to patients with milder forms of SDB. Fourth, there is a need to validate existing general patient-reported measures for patients with SDB and to develop additional patient-reported measures to evaluate the specific experiences of patients with SDB. The lack of an SDB-specific PREM remains an important gap within the literature: understanding elements of patient experiences specific to sleep-care may assist improvements to the design of alternative clinical care consistent with the principles of person-centred care. Lastly, the improvement over time may be a result of selection bias where patients who improved remained in the study while those who did not improve were lost to follow-up; approximately half of the patients dropped out over the course of the trial. Moreover, we did not perform imputation for missing data which may have limited statistical power to detect a difference between groups. As this study was undertaken in a clinical setting, we cannot conclude that the loss to follow-up was random or non-differential. Therefore, the results of the study should be interpreted with caution.

## Conclusion

In this paper, we showed that patient-reported outcomes improved over time in ACP-led clinics and that those improvements were not significantly different from improvements for patients receiving physician-led care. Considered with other research suggesting tangible clinical and economic impacts of ACP clinics for sleep disordered breathing, our analyses suggest adopting this model may allow for timely patient access while maintaining high quality of care.

## Electronic supplementary material

Below is the link to the electronic supplementary material.

Supplementary Material 1

Supplementary Material 2

## Data Availability

The data from the trial are not openly available for reasons of patient safety and privacy. They are available from the corresponding author on reasonable request.
