# Supplementary material for: Comparison of patient-reported outcomes between alternative care provider-led and physician-led care for severe sleep disordered breathing: secondary analysis of a randomized clinical trial
Source: J Patient Rep Outcomes. 2024 Sep 26;8:107. doi: 10.1186/s41687-024-00747-3 (PMC11427643; doi:10.1186/s41687-024-00747-3)
Supplement: Supplementary file 1 — Supplementary Material 1 [file 41687_2024_747_MOESM1_ESM.docx]

# Supplementary information for “Comparison of patient-reported outcomes between alternative care provider-led and physician-led care for severe sleep disordered breathing: secondary analysis of a randomized clinical trial”

**Supplementary methods information**. This section contains relevant information on measure selection and clinically significant improvements.

HUI-2 and HUI-3. The Health Utility Indexes measure health status by assessing similar and overlapping domains, but Mark 2 and Mark 3 assess distinct elements of health, as outlined in a review by Horsman and colleagues.^22^ For instance, while the HUI-2 emotion domain assesses “distress and anxiety”, the HUI-3 emotion domain assess “happiness versus depression”. While the HUI-2 cognitive domain assesses “learning, the HUI-3 cognitive domain assesses the “solving of day-to-day problems). While the HUI-2 pain domain assesses “frequency [and] control”, the HUI-3 pain domain assesses “severity”. When establishing the RCT, we included both Mark 2 and Mark 3 to capture additional data on patient-reported health status.

SAQLI. Flemons and Reimer note clinically meaningful change on both domain and total scores can indicate no change (difference of -1 to 1) or minimal important difference (difference of 1.1 to 3 or -1.1 to -3), moderate difference (difference of 3.1 to 5 or -3.1 to -5) and large differences (difference of 5.1 to 7 or -5.1 to -7).^28^

ESS. For the Eppworth Sleepiness Scale, respondents answer “How likely are you to doze off or fall asleep in the following situations, in contrast to feeling just tired?” for a series of activities in daily life on a scale from 0 (“would never to doze”) to 3 (“high chance of dozing”). The ESS score is the sum of the eight responses with a maximum score of 24 (more than 10 reflects subjective sleepiness). For patients with SDB, recent evidence suggests that the minimum clinically meaningful improvement is a fall in ESS score between 2 and 3.^31^
