# Supplementary material for: Comparison of patient-reported outcomes between alternative care provider-led and physician-led care for severe sleep disordered breathing: secondary analysis of a randomized clinical trial
Source: J Patient Rep Outcomes. 2024 Sep 26;8:107. doi: 10.1186/s41687-024-00747-3 (PMC11427643; doi:10.1186/s41687-024-00747-3)
Supplement: Supplementary file 2 — Supplementary Material 2 [file 41687_2024_747_MOESM2_ESM.docx]

**Supplementary information for reviewers: blinded protocol publication**

The following is a partial, direct excerpt of the publication of the trial protocol cited in the submitted manuscript. A description of the protocol is shared in-text (pages 6-7) but this provides a fuller description of full randomized control study.

As much of this document is unmodified text and from the published protocol, this document can theoretically be used to break the blinding by reviewers.

We entrust journal editors and staff with the decision of whether to share this information with reviewers.

**Abstract**

Despite the high prevalence of sleep-disordered breathing (SDB) and the significant health consequences associated with untreated disease, access to diagnosis and treatment remains a challenge. Even patients with severe SDB (severe obstructive sleep apnoea or hypoventilation), who are at particularly high risk of adverse health effects, are subject to long delays. Previous research has demonstrated that, within a sleep clinic, management by alternative care providers (ACPs) is effective for patients with milder forms of SDB. The purpose of this study is to compare an ACP-led clinic (ACP Clinic) for patients with severe SDB to physician-led care, from the perspective of clinical outcomes, health system efficiency and cost.

Methods and analysis

The study is a randomised, controlled, non-inferiority study in which patients who are referred with severe SDB are randomised to management by a sleep physician or by an ACP. ACPs will be supervised by sleep physicians for safety. The primary outcome is positive airway pressure (PAP) adherence after 3 months of therapy. Secondary outcomes include: long-term PAP adherence; clinical response to therapy; health-related quality of life; patient satisfaction; healthcare usage; wait times from referral to treatment initiation and cost-effectiveness. The economic analysis will be performed using the perspective of a publicly funded healthcare system.

Ethics and dissemination

Ethics approval was obtained from the [BOARD] (ID: REB13-1280) at the [INSTITUTION]. Results from this study will be disseminated through presentations at scientific conferences and publication in peer-reviewed journals.

Trial registration number

[HIDDEN]

**Objectives**

The study is a randomised, controlled, non-inferiority study evaluating the 3-month and 1-year outcomes of an ACP Clinic for patients referred with suspected severe SDB. Patients will be randomised to one of two treatment arms—standard management by sleep physicians or management by ACPs in the ACP Clinic. The study hypotheses are that, compared with a traditional physician-led approach, the ACP Clinic will:

- result in similar PAP treatment adherence, subjective and objective response to therapy, health-related quality of life (HRQL) and patient satisfaction 3 months and 1 year after treatment initiation;
- reduce the time from referral to treatment initiation;
- result in similar number of sleep physician visits or diagnostic tests during the first year of treatment initiation;
- be cost-effective during the first year of treatment initiation.

**Method and analysis**

**Study** **setting**

The [REDACTED] Sleep Centre is a publicly funded, tertiary academic sleep centre in [CITY, PROVINCE], with a catchment area of ∼2 million people in [PROVINCE] and [ADDITIONAL PROVINCE]. There are eight sleep specialist physicians at the [NAME] Sleep Centre (six respirologists, one psychiatrist and one neurologist).

The [NAME] Sleep Centre receives ∼2500 referrals annually, of which 60% are for SDB and 30% are for severe SDB. Twenty-five polysomnograms (PSG) and 75 HSAT are performed each week and all tests are interpreted by a sleep physician. All newly referred patients with suspected SDB undergo HSAT prior to the initial assessment by a sleep physician. Consistent with current [COUNTRY] guidelines, patients are prioritised based on severity of SDB on HSAT, medical comorbidity, daytime sleepiness and whether they work in a safety-critical occupation. Patients are assigned to sleep physicians based on the suspected diagnosis. Any physician at the [NAME] Sleep Centre may assess patients with uncomplicated OSA, but prior to this study, patients with suspected severe SDB were only scheduled to see respirologists. Patients may undergo PSG at the physician's discretion if the diagnosis is uncertain or if there is a concern about ambulatory PAP auto-titration.

Consistent with current clinical guidelines, patients with mild or moderate SDB may be offered PAP therapy or referral to a dentist for oral appliance therapy, depending on symptoms and patient preference. However, PAP is recommended as first-line therapy for all patients with severe SDB. Patients are also counselled on lifestyle modification (eg, weight loss, avoidance of excessive alcohol and sedative use) as appropriate. Conventional practice at the [NAME] Sleep Centre is that ACPs conduct follow-up assessments, either in the clinic or by telephone, guided by sleep physician-approved protocols. For patients with uncomplicated OSA, we have previously demonstrated that this is an effective follow-up model. The primary sleep physician could be reconsulted by the ACP as necessary; physician reassessment could occur by review of the case with the ACP or direct follow-up with the patient.

Alternative care providers All ACPs are respiratory therapists (RT) who have completed a 2-year accredited respiratory therapy training programme in [COUNTRY]. Completion of this programme includes attendance of 300 hours of classroom and laboratory-based learning on respiratory physiology and over 800 hours of education and supervised clinical experience with invasive and non-invasive mechanical ventilation in a variety of medical contexts.32 In addition, ACPs at the [NAME] Sleep Centre have at least 5 years of experience assessing and managing patients with SDB. All ACPs are registered with the provincial respiratory therapy professional college in [PROVINCE, COUNTRY], which regulates RTs based on established standards of practice and continuing education requirements.

The scope of ACP practice at the [NAME] Sleep Centre is defined by a physician-approved policy that complies with provincial regulations for Registered RTs. ACP activities include: initial education about PAP therapy; follow-up assessment of patients on PAP; and ordering of arterial blood gas and HSAT. ACPs can also make adjustments to PAP equipment, including humidity, ramp and expiratory pressure release settings and small pressure changes to CPAP without a physician prescription. Additionally, regular review of ACP protocols for the management of patients on PAP and education on respiratory and non-respiratory sleep disorders occur bi-weekly at ACP-focused case conferences.

**Eligibility criteria**

Patients who are referred to the [NAME] Sleep Centre will be eligible to participate in the study if they meet at least one of the following inclusion criteria:

- Respiratory Disturbance Index (RDI) ≥30 events/hour on HSAT;
- mean nocturnal oxygen saturation ≤85% on HSAT;
- suspected sleep hypoventilation syndrome, defined by an RDI ≥15 events/hour on HSAT and partial pressure of carbon dioxide ≥45 mm Hg on arterial blood gas while awake.
- Patients already on supplemental oxygen, in whom HSAT is insensitive for the diagnosis of OSA, will be recruited if the airflow channel on the HSAT indicates severe SDB or if the investigators determine that the clinical suspicion of severe SDB is high.

Patients will be excluded from the study if they have a suspected concomitant sleep disorder other than SDB such as insomnia or narcolepsy, have previously been treated with PAP therapy for SDB, have primary health insurance from outside [PROVINCE] due to difficulties in collecting healthcare usage data, or if they fail to provide consent to participate in the study.

**Randomisation and blinding**

Participants will be randomly assigned to either the standard management by a sleep physician or to the ACP Clinic with a 1:1 allocation using a computer generated randomisation schedule.

The allocation sequence will be concealed until the patient is eligible and consented to the study. The research associates will allocate patients according to the randomisation schedule and the booking clerk will schedule the patient with the appropriate provider according to the [NAME] Sleep Centre scheduling policies. The allocation of the participants will be stored in a database, which is only accessible to the research associate. If the patient prefers to be assessed by a particular provider at the time of initial scheduling, then the allocation would be discontinued and the patient would be excluded from the study.

The research associate and booking clerks will not be blinded to the study. The ACPs and sleep physicians, including investigators, will not be able to identify study patients as both groups will be conducting clinics comprised of a combination of study and non-study patients and there will be no other indications that the patient is a study participant. Owing to the nature of the study, the participants will not be blinded to the study.

**Intervention**

Patients randomised to standard management will receive usual care as described above (see the ‘Study setting’). Patients will undergo an initial assessment by a sleep physician, who will establish a management plan with the patient that may include PSG, initiation of therapy for SDB and/or clinical follow-up. Follow-up may be delegated to an ACP, who will manage patients within their scope of practice as defined in existing physician-approved protocols. As is routine practice at the [NAME] Sleep Centre, ACPs will be able to refer patients back to the primary sleep physician for persistent symptoms of SDB or management of clinical issues outside of their scope of practice.

Patients randomised to care in the ACP Clinic will have an initial assessment by an ACP, during which time the management plan will be established by the ACP and the patient. Since this patient population is medically complex, the management plan will be reviewed with a sleep physician immediately after the assessment. The sleep physician will meet briefly with the patient to review any additional medical or sleep-related concerns before confirming the proposed plan for further testing and/or treatment. The presence of a sleep physician in the clinic was also deemed important in case a patient was unstable at the time of assessment (eg, severe hypoxemia, decompensated respiratory failure). Follow-up will occur with the ACP to review test results, discuss and initiate treatment and to assess treatment response. The ACP will be able to refer patients back to the primary sleep physician as in the usual care group.

In both groups, as is standard practice at the [NAME] Sleep Centre, HSAT requisitions will be completed by physicians or ACPs and interpreted by a sleep physician. PSG requisitions and interpretation, and prescriptions for PAP therapy will be completed by the primary sleep physician.

**Outcomes**

A summary of study outcomes and when they will be measured is presented in table 1.

***TABLE 1***

Outcome measures collection points

|  | **Baseline** | **3 months** | **1 year** |
| --- | --- | --- | --- |
| Adherence to therapy   - PAP adherence |  | ✓ | ✓ |
| Daytime sleepiness   - Epworth Sleepiness Scale | ✓ | ✓ | ✓ |
| Health-related quality of life/utility score   - Health-Utilities Index - Sleep Apnoea Quality of Life Index | ✓ | ✓ | ✓ |
| Patient satisfaction   - Visit-Specific Satisfaction Instrument 9 |  | ✓ | ✓ |
| Demand for sleep provider visits and diagnostic testing   - Number of ACP visits - Number of sleep physician visits - Number of HSATs and PSGs - Physician time spent per patient during ACP clinic (time-in-motion study) | − | − | ✓−* |
| Healthcare usage   - Number of outpatient physician visits - Number of hospitalisations - Number of emergency department/urgent care visits |  |  | ✓ |
| Healthcare costs   - Healthcare usage costs - HSAT and PSG - Treatment costs |  |  | ✓ |
| Wait times   - Time from referral to initiation of therapy | − | − | −* |

*Outcomes will be collected throughout the study.

ACP, alternative care provider; HSAT, home sleep apnoea testing; PAP, positive airway pressure; PSGs, polysomnograms.

***End of TABLE 1***

**Primary outcome**. The primary outcome is PAP adherence after 3 months of therapy. Treatment adherence will be reported in terms of average nightly use and dichotomised based on whether patients used PAP therapy for at least 4 hours a night for at least 70% of nights. Adherence downloads from the preceding 4 weeks will be obtained from each patient's PAP machine.

PAP adherence was chosen as a primary outcome for several reasons. First, many outcome measures such as daytime sleepiness, quality of life or functional status are related to PAP use. In addition, many of the outcome measures used in other studies are subjective; patients often underestimate their symptoms leading to relative insensitivity of symptom scores as measures of treatment effectiveness.

Second, treatment of severe SDB with PAP is associated with reductions in the risk of cardiovascular disease, development of metabolic disorders such as diabetes, healthcare usage and mortality. These are important clinical outcomes for individual patients and for health system usage and cost. Given that the population under study is at particularly high risk for these adverse medical consequences, we determined that treatment adherence was of a higher priority than other outcomes for a comprehensive evaluation of this novel model of care for severe SDB.

Finally, PAP adherence is commonly used as an outcome measure in studies examining service delivery models for patients with SDB, and has been identified as an indicator of high-quality care for SDB.

**Secondary outcomes** A number of secondary outcomes, related to clinical effectiveness, healthcare usage, system efficiency and cost, will be analysed. Additional details on the secondary outcomes are available in the online supplementary material.

*[Redactor’s note: Details on the following have been restricted to the relevant outcomes for the current study]*

**Daytime sleepiness.** The Epworth Sleepiness Scale is a validated patient questionnaire assessing daytime sleepiness.

**Health-related quality of life (HRQL).** HRQL will be measured using general and disease-specific instruments. The Health-Utilities Index (HUI) and the short-form Sleep Apnoea Quality of Life Index (SAQLI) will be used to measure general HRQL and disease-specific HRQL, respectively.

**Patient satisfaction** The Visit-Specific Satisfaction Instrument (VSQ-9) is a validated measure of patient satisfaction with an outpatient visit.

**Statistical analysis**

This study will use a modified intention to treat analysis, in which results will be analysed for patients who are randomised and have treatment adherence data 3 months after initiating PAP therapy.

Paired t-tests will be used to compare clinical outcomes from baseline to 3 months and 1 year and unpaired t-tests will be used to compare time to treatment initiation and measures of patient demand. Multiple logistic regression will be used to identify predictors of study outcomes using variables identified as predictive on univariate regression. Outcomes will be transformed into binary variables based on clinically relevant cut-offs.

*[Redactor’s note: Details on the following have been restricted to the relevant outcomes for the current study]*

An estimate of mean utility scores based on the HUI questionnaire will be calculated and the average quality-adjusted life-year (QALY) will be calculated for each study arm.

All study outcomes will be analysed in different subgroups to clarify the impact of ACP-led care for patients with different patient flow pathways or clinical treatments. Prespecified subgroups include:

- patients who undergo PSG versus patients who do not undergo PSG;
- patients treated with bi-level PAP versus patients treated with CPAP;
- patients who are treated with oxygen versus patients treated without oxygen.

**Sample size**

The study is powered to assess the non-inferiority of ACP-led care compared with usual care by sleep physicians. A non-inferiority margin of −1 hour of PAP adherence was determined by consensus of the investigators and has been used in previous studies comparing different models of care for SDB. The study will require 138 patients (69 in each arm) to achieve 90% power with a type I error of 0.05, using this non-inferiority margin and a SD of 2 hours of nightly CPAP use.48 The SD used in the sample size calculation was based on the results of two previous studies. To account for withdrawals and loss to follow-up (∼15% at the [NAME] Sleep Centre), recruitment will continue until 3-month adherence data is available for 150 patients.

**Trial status**

Patient recruitment began in October 2014 and was completed in August 2016. We are in the process of collecting baseline measurements, 3-month and 1-year follow-up measurements and anticipate the completion of 3-month and 1-year data collection by December 2016 and December 2017, respectively.

**Ethics and dissemination**

The ethics approval process involved reviewing the study with respect to content and compliance with applicable research and safety regulations. In addition to the initial approval of the study, the [RESEARCH ETHICS BOARD NAME ACRONYM] will review the study on an annual basis. Any modification to the study protocol will require a formal amendment to the protocol and submitted to the [RESEARCH ETHICS BOARD NAME ACRONYM] for approval.

In addition, the study is registered under Clinicaltrials.gov (ID: [REDACTED]). Changes or updates to the study, including the study protocol, must be made at least every 12 months. Prior to the completion of the study, the record must be reviewed every 6 months.

The principal investigator and the attending physician are responsible for assessing, reporting and managing solicited and spontaneously reported adverse events and other unintended effects of trial interventions or trial conduct.

**Data quality and management**

To promote data quality, the research associates will randomly select 10% of eligible patients and review the data entered and collected. Range check for data values will be performed for the entire data set.

In accordance with research ethics board approval, all data will be stored on a secure network drive within [INSTITUTION] firewalls and will be accessed by the principal investigator and research associate using password-protected study computers through a Virtual Private Network. Identifying information will be replaced with a unique identifier in any data that is reviewed by investigators, with the associated identifying data stored in a separate password-protected file and accessible only to the research associate and principal investigator for the purposes of reconciling data errors. The clinical members of the research team may have access to clinical information on study patients as part of clinical care, but will not have access to identifiable patient records within the research database. The final data set, without any identifiable information, will be accessible to the principal investigator. The research team may access the data set on request.

**Consent and withdrawal**

Patients will be recruited by a research associate who is not involved in the clinical management of SDB. Written informed consent will be obtained from patients prior to any participation in the study. The research associates will contact patients prior to the 3-month and 1-year follow-up appointments to promote participant retention and complete follow-up.

Participants can withdraw from the study at any time without any consequences. At time of withdrawal, the participant will be asked if additional healthcare usage data can be collected. If the participant chooses not to participate in further data collection, data contributed up to the point of withdrawal will be retained but no further data will be collected.

**Dissemination**

Results will be disseminated through publications in peer-reviewed journals; one manuscript will report the 3-month clinical data, and a second manuscript will include longer term clinical and health system outcomes after 1 year. Publications from this research will add to the emerging literature on novel models of care for SDB, and in particular will address whether ACPs can be used to manage a more severe subset of patients. This study lays the foundation for additional research to explore the optimal way to deliver care in the context of such a highly prevalent disease.

The results of this study will also be shared with operational leaders at the [NAME] Sleep Centre, to determine whether the ACP Clinic model is effective for patients. We will also disseminate the protocol and the study findings to other centres within and outside of [COUNTRY] through the [COUNTRY-SPECIFIC NETWORK], and provide insights on our experience to those groups looking to implement such a programme.

**Discussion**

In many jurisdictions, providing timely access to care for patients with SDB is a challenge due to a shortage of sleep physicians. Building on the results of previous studies of alternative models of care delivery for OSA, this study aims to determine whether ACPs can manage patients with severe SDB. If the study aims are met, the role of ACPs in the management of severe SDB will be more clearly understood in terms of their impact on clinical, economic and health system outcomes. The results of this study will help sleep clinicians and health system administrators to determine the optimal scope of practice of ACPs.

As is the case in many healthcare systems with limited access, sleep clinics may adopt strategies that prioritise patients with severe disease above those with milder disease. This strategy allows patients who are at higher risk of medical complications to be assessed sooner and is particularly important when wait times are long. It could be argued that ACPs are best used to manage milder patients as has been demonstrated in previous studies, thus creating capacity for sleep physicians to assess severe patients. However, given the burden of SDB in the population, a significant proportion of whom have severe disease, and insufficient supply of sleep physicians in many jurisdictions, it is probable that delays for severe patients are also long. Furthermore, if ACP capacity could be increased, it is conceivable that wait times for less severe patients might paradoxically be shorter than for more severe patients. Thus, a strategy that aims to directly improve access for higher risk patients, such as using ACPs to manage patients with severe SDB, is preferable to one in which ACPs only assess patients who are of lower priority.

Many healthcare systems have insufficient resources to address this imbalance of supply and demand. This study proposes to mitigate this problem through the use of non-physician healthcare providers, under the hypothesis that ACPs can improve access and are cost-effective. However, when proposing a novel model of care, it is essential to demonstrate clinical effectiveness in addition to evaluating the impacts on the healthcare system. This study will comprehensively evaluate an ACP-led pathway for the management severe SDB, including clinical outcomes as well as demand for healthcare resources and costs.

**Limitations**

The proposed study has several limitations. First, it is possible that patient preference for care by a sleep physician may influence study outcomes. Our clinical experience with ACP care at the [NAME] Sleep Centre has been that patients do not object to this management pathway. Furthermore, a previous study of nurse-led care for OSA demonstrated that patient satisfaction did not suffer with non-physician management and in fact was higher with respect to certain aspects of the healthcare encounter. The research associate will reassure patients that ACP care will be supervised by a sleep physician and that referral back to a sleep physician can be initiated at any point at the patient's request. Finally, we have selected objective outcomes such as PAP adherence, wait times and healthcare costs to minimise potential confounding by patient perceptions of the care they receive.

A second potential limitation relates to the use of multiple instruments to collect patient reported outcomes. It is possible that patients will experience significant burden from these questionnaires, leading to incomplete or inaccurate data. However, previous work has demonstrated that ‘questionnaire fatigue’ is not significant in an outpatient setting. Additionally, we have performed a small time trial for our battery of instruments with patient engagement researchers and observed that the entire set of questionnaires takes ∼15 min to complete. Consequently, we do not anticipate that the completion of questionnaires will lead to any delays on the day of the visit. Our expectation of no significant adverse impact on clinic flow is consistent with previous literature.

Third, this study uses RTs as alternative care providers. RTs are highly trained in respiratory care and ventilation but are not a recognised health profession in some parts of the world. Thus, the study results might not be generalisable to jurisdictions in which the RT role does not exist. We recognise that an ACP in any clinical setting should have expertise in managing the patient population; in this regard, only RTs with additional expertise in the management of SDB are employed at the [NAME] Sleep Centre. Similarly, nurses and other healthcare professions should be comfortable with respiratory care and ventilation, and may require specific additional training to achieve this.

Finally, patients who are randomised to the ACP Clinic arm could experience an adverse outcome related to ACP care, such as the need for an emergency department visit or hospitalisation due to unrecognised illness at the time of assessment. This important patient safety risk will be mitigated by the assignment of each ACP Clinic patient to a primary sleep physician, who will review the management plan with the ACP and meet each patient at the time of the clinic visit. Furthermore, our experience with the delegation of follow-up care to ACPs at the [NAME] Sleep Centre suggests that trained RTs appropriately identify complex patients, and obtain guidance either through direct communication or by scheduling a follow-up clinic visit with the sleep physician. While we have mandated physician supervision during the ACP Clinic, we recognise that in other jurisdictions, advanced practice nurses or nurse practitioners may have the expertise to manage these patients independently.
